# The intangible benefits of vaccination – what is the true economic value of vaccination?

**DOI:** 10.3402/jmahp.v3.26964

**Published:** 2015-08-12

**Authors:** Paolo Bonanni, Juan José Picazo, Vanessa Rémy

**Affiliations:** 1 Department of Health Sciences, University of Florence, Florence, Italy; 2 Department of Clinical Microbiology, Hospital Clinico San Carlos, Madrid, Spain; 3 Sanofi Pasteur MSD, Lyon, France

**Keywords:** vaccination, economic evaluation, intangible benefits, healthcare system, antibiotic resistance, complications

## Abstract

Previous economic evaluations of new vaccines largely focussed on a narrow set of benefit categories, including primarily health gains and disease-related medical cost-savings, which probably resulted in underestimates of the true value of these vaccines. Other economic benefits of vaccines could be considered to assess the full economic value of vaccination, such as, for example, impact of the human papillomavirus vaccine on women's fertility through the decrease in precancerous lesions and, therefore, in the number of diagnostic and treatment interventions, which can be associated with an increased risk of subsequent pregnancy complications. Vaccines’ impact on resource allocation at hospital level or on antimicrobial resistance, such as pneumococcal conjugate vaccines that have substantially reduced infections due to antimicrobial non-susceptible strains, thereby rendering the residual disease easier to treat, are other examples of intangible benefits of vaccination. These benefits are generally not considered in economic evaluations because they may not be immediately visible and are difficult to quantify. However, they should be taken into consideration in health technology assessments to enable those responsible for healthcare policies to make well-informed decisions on vaccination.

National policymakers and international organisations commonly use the results of economic evaluation in the frame of their health technology assessments to inform spending decisions on vaccination programmes. However, existing economic evaluations tend to focus on a narrow set of benefits with a narrow perspective, including primarily health gains and disease-related medical cost-savings. They do not usually consider the broader benefits, such as outcome-related productivity gains (i.e., improved economic productivity due to prevention of diseases and associated mental and physical disabilities), impact on healthcare system efficiency, or some positive externalities (i.e., prevention of antibiotic resistance). One of the reasons for not considering some of these benefits is that many of them are ‘invisible’ and/or difficult to quantify. Some economic benefits related to outcome productivity gains have been discussed in other papers in this special issue (1, 2). The objective of this article is to highlight some intangible benefits of vaccination that are usually neglected in traditional economic evaluations, and which could contribute to a more accurate assessment of the full value of vaccination.

## HPV vaccination impact on fertility and neonatal morbidity

The first example of a neglected benefit of vaccination is the impact of human papillomavirus (HPV) vaccination on women's fertility, and neonatal prematurity. Cervical intraepithelial neoplasia (CIN) is the precursor of invasive cervical cancer and is due to HPV infection. Conisation, a standard treatment for high-grade CIN, is associated with an increased risk of subsequent pregnancy complications, such as premature delivery and possible subsequent life-long disability. Although HPV vaccination has the potential to decrease neonatal morbidity and mortality, this has not been taken into account in published cost-effectiveness models (3).

A recent German study estimated that if HPV 16/18 vaccines were to be considered as ‘vaccines against conisation-related neonatal morbidity and mortality’ only (i.e., not considering vaccination's impact of cervical cancer), they would have the potential to be cost-effective (4). This adds to the well-recognised benefits of HPV vaccination on cervical cancer and CIN. Future cost-effectiveness studies should take this significant benefit into account when assessing the economic value of HPV vaccination to ensure that policymakers have accurate and relevant information to reach well-founded decisions regarding HPV vaccination programmes.

## Reduction in disease severity, complications, and comorbidities

Vaccination prevents people from being infected and, thus, developing disease but it can also prevent severe complications. For example, there is increasing evidence supporting the use of influenza vaccination for the secondary prevention of myocardial infarction, which is now usually accounted for in economic evaluations 5,6. However, these benefits are not yet considered for two other vaccines which could also potentially reduce myocardial infarction and stroke risk in those aged ≥50 years:

- *Herpes zoster (HZ) vaccine against shingles*: it has been reported that people who develop shingles are at about a 30% higher risk for stroke (7).
- *Pneumococcal vaccines*: the results from a large hospital-based case–control study suggest that pneumococcal vaccination was associated with a 50% lower risk of myocardial infarction 2 years after vaccination (8).

Recently, the World Health Organization concluded that measles vaccination could be associated with large reductions in all-cause childhood mortality (9). This is supported by results from a recent study analysing pre- and post-vaccination data in Denmark, UK, and United States, providing evidence for a generalised prolonged (2–3 years) impact of measles infection on subsequent mortality from other infectious diseases, thus suggesting that measles vaccination might also produce strong and durable herd protection against all-cause infectious diseases (10). This implies that mortality and morbidity reductions linked to measles vaccination might be much greater than previously considered and reinforce the importance of measles vaccination in a global context.

Lastly, vaccines may also reduce severity of infection and thereby the amount of healthcare resources used. Disease can occur in vaccinated individuals as vaccines are usually not 100% efficacious; however, in such cases, the disease is usually milder than in non-vaccinated individuals (11). For example, in a German efficacy study of an acellular pertussis vaccine, vaccinated individuals who developed whooping cough had a significantly shorter duration of chronic cough than controls (12).

## Reduction of pressure on healthcare systems

Research highlights the importance of hospitals being able to cope in the event of unexpectedly large demands, for example, from an epidemic outbreak of an infectious disease such as swine influenza (13). This results in capacity being held back in preparation for such events, which represents around 5% of the total cost of an emergency admission (14).

In addition to reducing healthcare costs, vaccination can help to strengthen the sustainability of healthcare systems, especially at the hospital level. For example, vaccines such as influenza and rotavirus vaccines can contribute to a reduction in hospital admissions, thereby enabling a better allocation of resources.

In temperate climates, rotavirus gastro-enteritis (RVGE) coincides with other common childhood epidemics, causing more than 40% of the total burden of infant hospitalisations (i.e., respiratory syncytial virus, influenza) occurring over the same seasonal period, leading to the so-called ‘winter chaos’ (15). Coincidence of these epidemics places healthcare systems under pressure, causing an increased risk of nosocomial infections from periodic overcrowding, resulting in additional cases, adverse events, understaffing, lack of beds due to extended hospital stays, and closure of wards to new admissions (16–18). Additional costs resulting from the coincidence of RVGE with other infections are likely to occur in a number of areas that may not be fully captured in cost-effectiveness analyses. Furthermore, the increased burden on hospital capacity can put pressure on staff, affecting their ability to deliver high-quality care to children or delaying planned surgeries for other children (18). Post-vaccination surveillance data have shown a delay in the onset of RVGE epidemics, leading to a reduction in the overall numbers of cases and in the epidemiological overlap, which may decrease workload pressures. For example, in Belgium, following the introduction of rotavirus vaccination, there was a 65–83% reduction in rotavirus hospitalisations and a delay of 4–6 weeks in the onset and peak of the RVGE epidemic (19, 20). Another Belgian study in one paediatric hospital evaluated the potential difference between pre- and post-vaccination periods in hospital pattern and personnel management. The results indicated that bed-day occupancy, bed-day turnover, and unplanned readmissions for acute gastroenteritis were lower in the post-vaccination compared with the pre-vaccination periods, suggesting an improvement in quality of care and a reduced pressure on hospital resources after rotavirus vaccination introduction (21).

The pressure on resources induced by RVGE or influenza for hospitals and emergency departments and the resultant impact on budgets and the ability to deliver care may be underestimated. While disease-related hospitalisation costs are usually included in economic studies, the impact of rotavirus infections on healthcare systems and hospital workload and organisation due to excess nosocomial infections, increased hospital resources, and hospital disruption are not usually considered.

## Contribution of vaccination to antimicrobial resistance

Another example of ‘invisible’ benefits is the role that vaccination can play as part of institution-wide antimicrobial stewardship programmes. Antimicrobials have played an important role in the control of infectious diseases. However, microorganisms are able to mutate, and some of these mutations confer resistance to the antimicrobials. The continuous use of these drugs can lead to the selection of resistant strains, thereby rendering the antimicrobial ineffective. This antimicrobial resistance is a serious threat and leads to increasing healthcare costs, longer hospital stays, treatment failures, and even deaths. In 2009, the ECDC and the EMA estimated that 25,000 Europeans die each year as a direct consequence of a multidrug-resistant infection, with costs estimated at €1.5 billion per year (22). Therefore, any strategy that can reduce the use of antibiotics should be considered as important in a long-term portfolio of measures to combat this global health issue. Both viral and bacterial vaccines have the potential to reduce the community's need for antibiotics. Vaccines reduce the prevalence of infection and, subsequently, the prevalence of disease in the population which, in turn decreases the use of antibiotics. Viral vaccines will reduce the number of viral infections that are erroneously diagnosed as bacterial infections, and thus wrongly treated with antibiotics. Furthermore, many viral infections predispose patients to secondary bacterial infection, which, in the past, often lead to antibiotics being prescribed for the prophylactic prevention of these secondary infections. This is a well-known problem for chickenpox and influenza, for which effective vaccines exist. In the case of chickenpox, one of the most common pathogens for secondary infection is *Staphylococcus aureus* and of particular concern are infections caused by the methicillin-resistant strains (methicillin-resistant *Staphylococcus aureus*), which are responsible for an estimated 150,000 infections every year in the European Union alone (23). Pneumococcal conjugate vaccination (PCV) has been reported to reduce invasive pneumococcal disease caused by antimicrobial non-susceptible vaccine types by 81% in children aged ≤2 years (24). Although in the case of PCV7 vaccine, there was a concomitant increase in disease caused by non-vaccine types, such as the antimicrobial non-susceptible strain, 19A, this did not reverse the overall reduction in disease caused by non-susceptible strains. The inclusion of PCV13 decreased the incidence of serotype 19A, very aggressive and resistant to antimicrobials (25).

A recent review reported that all identified studies showed decreased antibiotic use associated with initiation of vaccination programmes or increased uptake of available vaccines (mainly influenza and pneumococcal vaccines). Reductions in antibiotic use ranged from 5 to 10% in randomised controlled trials, to relative reductions of 64% in epidemiological studies, suggesting that vaccination programmes may reduce antibiotic utilisation and, consequently, antibiotic resistance (26).

In the UK, the Joint Committee on Vaccination and Immunisation noted that whilst new antibiotics and greater stewardship of existing antibiotics in hospitals and primary care were essential, vaccination was equally important in the strategy, by reducing the exposure of the population to potentially unnecessary antibiotic therapy. The committee, therefore, advised that future cost-effectiveness analyses of vaccine programmes should account for the potential benefits associated with reducing antimicrobial use if possible (27).

## Value of vaccination in elderly and polymedicated population

While economic evaluations of vaccines always compare the total costs of a new vaccination programme with the total costs of the current intervention, few evaluations consider the broader benefits of vaccines versus the use of medical treatments such as drugs. Indeed, vaccines not only lead to a reduction in treatment costs by preventing disease, they can also avoid problems associated with long-term treatments and polymedication, particularly in the elderly. For example, patient drug non-adherence rates, especially in the elderly, have been estimated to be between 25 and 75%, and the lack of adherence is estimated to cost European governments about €125 billion per year (28, 29). As discussed in another article in this special issue, the current increase in the population aged ≥60 years leads to the conclusion that, by 2050, the number of older persons (>60) in the world will exceed the number of young people (<15) for the first time in history (2, 30). The elderly often have multiple comorbidities, which, combined with immunosenescence, result in an increased susceptibility to many infectious diseases, and in poorer outcomes after infections which are also strongly associated with unhealthy life style, dietary deficiency, and polymedication (31, 32). Polymedication, which is very frequent in the elderly and patients with chronic conditions, can lead to an increased risk of adverse events or a decrease in treatment efficacy due to potential interactions between prescribed and/or non-prescribed drugs. Systemic or cognitive adverse effects of drugs may also be amplified in older adults, leading to confusion, falls, cardiovascular, or respiratory events. These issues result not only in health consequences to patients but also in high costs to healthcare systems. A recent review suggested that the economic impact of administration errors, inappropriate drug prescription, and poor adherence in elderly patients is substantial, with hospital costs being the main driver (33).

Common infectious diseases in the elderly, such as influenza, pneumococcal infections, or HZ, can be challenging to manage and lead to complications that may contribute to their overall functional decline. As an example, patients with post-herpetic neuralgia (PHN), the most frequent chronic complication of HZ, are often of an advanced age and are likely to have more than one comorbidity, such as cardiovascular disease, diabetes, and chronic obstructive pulmonary disease, for which they receive several medications (34). PHN management frequently requires prolonged combination therapy and various conditions such as renal and hepatic impairment, cardiovascular, cerebrovascular and respiratory disease, and psychiatric conditions may modify the efficacy or the tolerability of prescribed drugs. Therefore, patients should be regularly monitored to assess response to treatment, adverse effects, negative impact on comorbidities, and functional decline (34). Prevention of HZ and PHN with vaccination could offer an effective solution to avoid increasing their risk for adverse events and functional decline.

Although issues, such as non-adherence or potential side effects, may also occur with vaccines’ administration, the limited number of required doses and their good safety profile limit these risks and associated costs. While these issues potentially minimise patients’ benefits through reduced efficacy, potential adverse events, and accelerated functional decline, they represent an additional medical and economic burden that is not usually taken into consideration in economic evaluations of vaccination, in particular in older and polymedicated populations.

## Conclusions

Economic evaluations do not usually take into consideration the lost opportunity for economic growth or savings that can be achieved if broader diseases’ complications and comorbidities are prevented or if resource allocation within the healthcare system is improved. It is believed that if policymakers were to include the appropriate factors for avoiding disease altogether (the intangible benefits of health) in the calculation, the value currently attributed to vaccines would be seen to be underestimated by a factor between 10 and 100 (35).

Broader perspectives may require more extensive data collection. For example, analysis of the economic effects of vaccination on antibiotic resistance will require information that may not be currently available. Evidence on the broader benefits of many health interventions is also very sparse. Understanding the complex relations between health interventions, health outcomes, education, and labour productivity has implications for all types of interventions.

Thus, although some intangible benefits of vaccination may be difficult to quantify, they should be considered in Health Technology Assessments to enable policy makers making well-informed decisions regarding vaccination, taking into account all the benefits for health, healthcare systems, and the wider economy, simultaneously with the cost of vaccination.
